# *MNS1* variant associated with *situs inversus* and male infertility

**DOI:** 10.1038/s41431-019-0489-z

**Published:** 2019-09-18

**Authors:** Joseph S. Leslie, Lettie E. Rawlins, Barry A. Chioza, Oluwaseun R. Olubodun, Claire G. Salter, James Fasham, Hannah F. Jones, Harold E. Cross, Simon Lam, Gaurav V. Harlalka, Martina M. A. Muggenthaler, Andrew H. Crosby, Emma L. Baple

**Affiliations:** 1 Institute of Biomedical and Clinical Science, RILD Wellcome Wolfson Centre, University of Exeter Medical School, Royal Devon & Exeter NHS Foundation Trust, Barrack Road, Exeter, EX2 5DW UK; 2 Peninsula Clinical Genetics Service, Royal Devon & Exeter Hospital, Gladstone Road, Exeter, EX1 2ED UK; 3 Department of Ophthalmology, University of Arizona College of Medicine, Tucson, AZ USA; 4 Department of Cardiology, Royal Devon and Exeter NHS Foundation Trust (Wonford), Barrack Road, Exeter, EX2 5DW UK

**Keywords:** Genetics research, Disease genetics

## Abstract

Ciliopathy disorders due to abnormalities of motile cilia encompass a range of autosomal recessive conditions typified by chronic otosinopulmonary disease, infertility, situs abnormalities and hydrocephalus. Using a combination of genome-wide SNP mapping and whole exome sequencing (WES), we investigated the genetic cause of a form of *situs inversus* (SI) and male infertility present in multiple individuals in an extended Amish family, assuming that an autosomal recessive founder variant was responsible. This identified a single shared (2.34 Mb) region of autozygosity on chromosome 15q21.3 as the likely disease locus, in which we identified a single candidate biallelic frameshift variant in *MNS1* [NM_018365.2: c.407_410del; p.(Glu136Glyfs*16)]. Genotyping of multiple family members identified randomisation of the laterality defects in other homozygous individuals, with all wild type or *MNS1* c.407_410del heterozygous carriers being unaffected, consistent with an autosomal recessive mode of inheritance. This study identifies an *MNS1* variant as a cause of laterality defects and male infertility in humans, mirroring findings in Mns1-deficient mice which also display male infertility and randomisation of left–right asymmetry of internal organs, confirming a crucial role for MNS1 in nodal cilia and sperm flagella formation and function.

## Introduction

Cilia and flagella are highly conserved and important protuberances of the plasma membrane that perform a diverse range of functions in the human body. Flagella are larger organelles that have a similar ultrastructure to cilia and function to propel spermatozoa. Motile cilia beat rhythmically to induce the movement of fluids, whereas non-motile or primary cilia functions in sensory perception and cell signalling [1]. Nodal cilia are expressed transiently during embryonic development in vertebrates and establish left–right laterality [2], although the precise mechanisms that underlie this function remain poorly understood. Previous studies have directly correlated defective nodal cilia with situs abnormalities in mouse models [2, 3].

Cilia are present on almost all cell types and over 2000 proteins are suspected to contribute to the ciliary proteome [4]; as a result disorders of ciliary function, termed ciliopathies are genetically heterogeneous. A diverse range of phenotypical outcomes are associated with ciliopathies including renal cyst formation, retinal degeneration, hearing loss, cerebellar anomalies, polydactyly, skeletal dysplasia, bronchiectasis, male infertility and situs abnormalities [1, 5]. The specific clinical features are determined by the sub-type, function, distribution and specific mutation of the cilia that are affected. Abnormalities of motile cilia formation and function are associated with primary ciliary dyskinesia (PCD) and over 38 genes have been implicated in the disorder (OMIM PS244400) [6], which typically shows autosomal recessive inheritance. The clinical features of PCD include, chronic respiratory tract disease and male infertility caused by abnormal spermatozoa motility; between 40 and 50% of individuals with PCD exhibit *situs inversus* (SI) *totalis* and over 6% exhibit complex situs abnormalities, including heterotaxy and associated congenital heart disease [7].

The molecular defects that underlie laterality defects alone, without other features of ciliopathies are more complex and include disruption of ciliary proteins (*CFAP53* [8] and *PKD1L1* [9]) leading to dysmotility of the nodal cilia and proteins not directly associated with cilia (*CFC1* [10], *ACVR2B* [11], *NODAL* [12], *MMP21* [13] and *ZIC3* [11]) acting through Nodal and Notch1 signalling pathways.

Meiosis specific nuclear structural 1 protein (MNS1) is expressed in human bronchial epithelium [14] and during spermatogenesis [15] and has previously been reported to have an essential role in motile ciliary function and sperm flagella assembly in mice [16]. A recent report by Ta-Shma et al. identified biallelic *MNS1* variants as a likely genetic cause of SI and male infertility [17]. Here we confirm this finding and identify a key role for MNS1 function in determining left–right body asymmetry and sperm flagella formation and function in humans by defining a homozygous founder frameshift variant in *MNS1*, in association with randomisation of left–right body asymmetry and male infertility, in four inter-related families of Amish descent, identified through a combination of homozygosity mapping and whole exome sequencing (WES).

## Materials and methods

Blood/buccal samples were obtained with informed consent (University of Arizona IRB—1000000050). DNA was extracted using standard techniques. Medical records for all SI affected individuals in the study were reviewed. All genotyped individuals underwent echocardiography and symptom review for PCD using the PICARD tool. Single-nucleotide polymorphism (SNP) genotyping was performed (HumanCytoSNP-12 v2.1 beadchip array, Illumina). WES (Illumina HiSeq) involved: Agilent Sureselect Whole Exome v6 targeting, read alignment (BWA-MEM (v0.7.17), mate-pairs fixed and duplicates removed (Picard v2.15.0), InDel realignment/base quality recalibration (GATK v3.7.0), single-nucleotide variant (SNV)/InDel detection (GATK HaplotypeCaller), annotation (Alamut v1.8) and read depth (GATK DepthOfCoverage). Dideoxy sequencing of *MNS1* and common Amish PCD founder variants, including *DNAH5* (NM_001369.2:c.4348 C>T; p.(Gln1450Ter) and NM_001369.2:c.10815delT; p.(Pro3606Hisfs)), *DNAI1* (NG_008127.1 (NM_012144.3):c.48 + 2_3insT; p.(Ser17ValfsTer12)), *DNAAF5* (NM_017802.3:c.2384 T>C; p.(Leu795Pro)) and *HYDIN* (NM_017558.4:c.2047 G>T; p.(Glu683*)) was undertaken using standard techniques. The *MNS1* founder variant was submitted to ClinVar (www.ncbi.nlm.nih.gov/clinvar, accession SCV000920769).

## Results

**Fig. 1:**
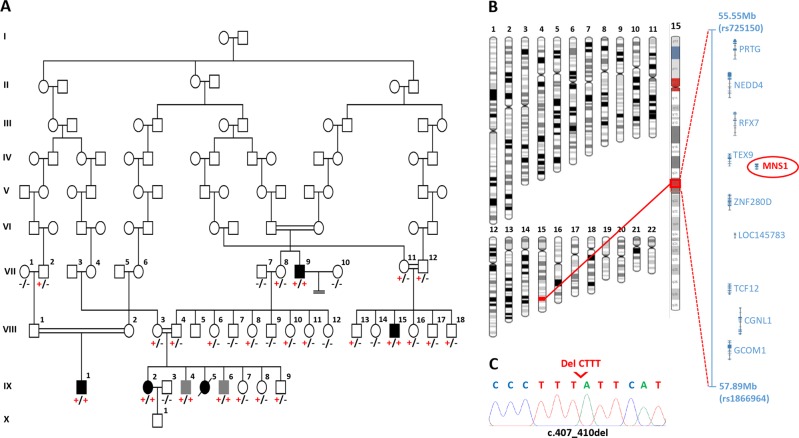
Biallelic *MNS1* NM_018365.2:c.407_410del p.(Glu136Glyfs*16) variant identified in an extended Amish family with laterality defects and male infertility. **a** Simplified pedigree of the extended Amish family investigated. Genotype is shown in red under individuals (+, mutant; –, wild type). All individuals affected by SI or male infertility were shown to be homozygous for the *MNS1* NM_018365.2:c.407_410del p.(Glu136Glyfs*16) variant (black symbols). Genotyping of additional family members identified randomisation of the laterality defect in homozygous individuals (grey symbols), with all wild type or heterozygous carriers being unaffected. **b** Visual depiction of the single ~2.34 Mb autozygous region on chromosome 15q21.3 (shown in red) common to all four SI affected individuals investigated and containing ten genes including *MNS1*. **c** Electropherograms showing the DNA sequence at the position of the *MNS1* NM_018365.2:c.407_410del variant (del CTTT) in a homozygous affected individual

Three males and one female aged 18–74 years from an extended Ohio Amish pedigree of four nuclear families were identified to have SI without symptoms of PCD (Fig. 1a). IX-5 died from meningitis aged 5 months and was discovered to have heterotaxy, subsequently a diagnosis of SI was made in IX-2, IX-1, VIII-15 and VII-9. All affected individuals were clinically assessed, no PCD symptoms were reported, with the exception of IX-1 who suffered recurrent otitis media. Semen analysis from VII-9 confirmed infertility, males IX-1 and VIII-15 were unmarried thus fertility was not determined, female IX-2 has no fertility problems (Table 1). All known genetic causes of PCD and/or situs abnormalities in the Amish were excluded in affected family members. Assuming that an autosomal recessive founder variant was responsible for the condition, genome-wide SNP genotyping was undertaken using DNA from four individuals with SI (VII-9, VIII-15, IX-1 and IX-2). This identified a single 2.34 Mb shared region of homozygosity on chromosome 15q21.3 (delineated by markers rs725150–rs1866964, chr15:g.55550578–57890256 [hg38]), containing ten genes including *MNS1* (Fig. 1b), no other notable regions of autozygosity (>1 Mb) were observed. WES was performed in parallel on DNA from VIII-15, IX-1 and IX-2 and no candidate variants in genes known to cause PCD or SI phenotypes (not yet described in the Amish) were identified. After filtering for call quality, variants predicted to have a functional consequence were analysed, identifying only a single candidate homozygous variant in the *MNS1* gene located within the chromosome 15 putative disease locus. This variant NM_018365.2:c.407_410del; p.(Glu136Glyfs*16), Chr15:g.56446887_56446890del [hg38] in *MNS1,* predicted to result in a frameshift and premature stop codon, was not listed in the Genome Aggregation Database (gnomAD), ClinVar, National Center for Biotechnology Information or the Human Gene Mutation Database (HGMDpro) and represented the only candidate shared variant genome-wide that could not be excluded on the basis of mode of inheritance or presence at high frequency in gnomAD (>2.5%). All four individuals with SI were confirmed to be homozygous for the *MNS1* variant by dideoxy sequencing (Fig. 1c). A total of 26 additional family members from multiple nuclear families within the extended Amish pedigree, linking back to an 8th generation common ancestor, were genotyped and clinically assessed (Fig. 1a). Co-segregation of the *MNS1* variant identified two additional male individuals (IX-4 and IX-6) who were homozygous, but did not exhibit SI (Fig. 1a; Table 1); one individual was too young for fertility analysis and the other had no children, but was unmarried and not assessed for fertility. Absence of dextrocardia was confirmed in the remaining 24 unaffected family members.

**Table 1:** Clinical features of individuals homozygous for *MNS1* NM_018365.2:c.407_410del; p.(Glu136Glyfs*16)

ID	Sex	Age (years)	Laterality defects	Dextrocardia	PCD symptoms	Fertility	Congenital malformations	MNS1 genotype
VII-9	M	74	*Situs inversus*	✓	Nil	Infertile	Nil	+/+
VIII-15	M	39	*Situs inversus*	✓	Nil	N/A (no children)	Nil	+/+
IX-1	M	18	*Situs inversus*	✓	Recurrent otitis media	N/A (no children)	Nil	+/+
IX-2	F	27	*Situs inversus*	✓	Nil	Fertile	Nil	+/+
IX-4	M	24	Nil	×	Nil	N/A (no children)	Nil	+/+
IX-5	F	5 m	Heterotaxy	✓	N/K	Deceased aged 5 m	Asplenia	N/K
IX-6	M	11	Nil	×	Nil	N/A	Nil	+/+

A comparison of the clinical findings of individuals within an extended Amish family identified as homozygous for *MNS1* NM_018365.2:c.407_410del; p.(Glu136Glyfs*16)

*M* male, *F* female, ✓ present, ×  not present, *N/K* not known, *N/A* not assessed, *m* months, *TGA* transposition of the great arteries

Nine heterozygous carriers of the *MNS1* variant were identified in a total of 225 Amish control samples (allele frequency: 0.02), not unexpected for a disease causing variant within this founder population.

## Discussion

Here we provide extensive co-segregation data and comprehensive clinical information of a homozygous *MNS1* Amish founder gene variant [NM_018365.2:c.407_410del; p.(Glu136Glyfs*16)] as responsible for SI and/or male infertility. Importantly the results of our study demonstrate the randomisation of left–right laterality development in individuals with biallelic pathogenic *MNS1* variants as opposed to a direct reversal; by genotyping and clinically investigating a total of 26 individuals within four inter-related Amish families. Six individuals were found to be homozygous for the *MNS1* NM_018365.2:c.407_410del variant, of whom four (67%) exhibited SI and two (33%) exhibited *situs solitus*. This randomisation of left–right asymmetry observed in homozygotes is consistent with findings associated with other motile ciliary disorders and closely mirrors the studies of *Mns1-*deficient mice, undertaken by Zhou et al. [16] who observed SI in eight of 36 (22%), left isomerism (heterotaxy) in a further six (17%) and *situs solitus* in the remaining 22 (61%) mice. Currently, the mechanisms underlying heterotaxy versus the SI phenotype amongst individuals with the same underlying cause of disease remain unclear [18]. Interestingly, Boshen et al. identified that *Mns1*-deficient mice were more susceptible to prenatal alcohol exposure which resulted in an increased rate of midline defects, leading to the suggestion that environmental factors might contribute to determining the type of laterality defects that occur in humans [19].

Importantly, our findings support those of a recent report by Ta-Shma et al. identifying *MNS1* variants as likely candidate causes of SI and male infertility in the absence of chronic otosinopulmonary symptoms. This study investigated four affected individuals from three pedigrees (from Palestine, Turkey and Jordan) and identified the same homozygous *MNS1* nonsense variant (p.(Arg242*)) in all four affected individuals [17]. However, unfortunately it was not possible to genotype other affected and unaffected family members (excepting the unaffected parents in one Palestinian family, who were heterozygous for the variant). As such, it is difficult to conclusively determine whether this represents the same founder pathogenic variant responsible for SI, or an otherwise rare benign regional variant. Ta-Shma et al. also investigated an Israeli family comprising of three nuclear families interlinking into a single pedigree with five individuals affected with PCD, four of whom were found to have SI, fertility was not determined. Incredibly both an *MNS1* NM_018365.2:p.(Gln203*) and a *DNAH5* p.(His4478Alafs3*) variant were identified and all affected family members were homozygous for the *DNAH5* variant, a known genetic cause of PCD and SI. The presence of the likely deleterious *DNAH5* gene variant rendered it difficult to interpret the relevance of the *MNS1* frameshift variant, present in homozygous form in only a single individual. However, the findings of Ta-Shma et al. when considered alongside our data and the findings in *Mns1* knockout mice are mutually supportive and conclusively confirm an essential role for MNS1 function in determining left–right body asymmetry and sperm flagella formation and function.

IX-5 is the only affected Amish individual identified in our study with heterotaxy (unfortunately no genotype is available), all other Amish individuals with laterality defects exhibited SI. Similarly all individuals with biallelic *MNS1* variants identified by Ta-Shma et al. had SI, except one who also had congenital heart defects. When considered with the range of laterality phenotypes seen in *Mns1* knockout mice, it seems likely that a range of laterality defects may be associated with disruption of MNS1 in humans.

The importance of nodal cilia in the determination of left–right asymmetry has been well established [20]. Although the exact mechanism is yet to be fully understood, all proposed models concur that nodal flow is essential for correct establishment of left–right asymmetry and is generated by the beating of nodal cilia between embryonic day (E)7.5 and E8.5 [21]. Ta-Shma et al. found *Mns1* expression strongly enriched in the ventral node of mouse embryos at E8.25, suggesting that *Mns1* may be a functional component of motile nodal cilia. Taken together with these findings, our results suggest that loss of MNS1 function may be sufficient to disrupt nodal flow, thus randomising the development of left–right asymmetry and causing situs abnormalities.

Of the five male individuals homozygous for the *MNS1* NM_018365.2:c.407_410del variant identified within our cohort, one was confirmed to be infertile, although further fertility assessment was not possible. Ta-Shma et al. reported two cases of male infertility in individuals homozygous for loss-of-function variants in *MNS1* [17]; semen analysis identified severely reduced flagellar motility and abnormal sperm morphology. *Mns1* has previously been reported to be highly expressed in mouse testis [15] and more recently, Zhou et al. found that male *Mns1*-deficient mice were sterile with immotile sperm and an epididymal sperm count of only 8% of wild type [16]. The latter study also reported that *Mns1* localised to, and formed an integral part of, sperm flagella; a finding supported by Ta-Shma et al. who reported localisation of *Mns1* predominantly in the mid and principal piece [16, 17].

Previous studies in *Mns1-*mice identified partial outer dynein arm (ODA) abnormalities in respiratory cilia, leading the author to suggest the implication of *MNS1* in PCD [16]. Ta-Shma et al. identified subtle ODA abnormalities in *MNS1*-deficient humans [17], however no individuals with biallelic variants in *MNS1* alone have been shown to have features of PCD. Interestingly, analysis of the respiratory cilia from the single individual whom Ta-Shma et al. determined was homozygous for both a pathogenic *DNAH5* variant and *MNS1* (NM_018365.2:c.607 C>T; p.(Gln203*)), revealed absence of the residual projections on the doublet microtubules of the ODA docking complex in addition to the absent ODAs typically caused by *DNAH5* variants. Ta-Shma et al. concluded that these findings alongside their co-immunoprecipitation and yeast two hybrid analyses indicate that MNS1 dimerizes and interacts with CCDC114, an ODA docking complex component, suggesting a crucial role for MNS1 in ODA docking complex assembly, our genetic findings add weight to this suggestion.

In summary, our study identifies a novel frameshift variant NM_018365.2:c.407_410del in *MNS1* in association with SI and male infertility, without other features of PCD or ciliopathies. Taken together with the findings of previous mouse and human studies, our data provide conclusive evidence for the importance of MNS1 in normal motile cilia function, determining left–right asymmetry and in the assembly and motility of sperm flagella. However, the precise molecular role of MNS1 in nodal cilia and sperm flagella formation and function remains unclear and further functional studies are required to fully characterise this.
